# Supplementary material for: Professionals’ experiences of what affects health outcomes in the sick leave and rehabilitation process—A qualitative study from primary care level
Source: PLoS One. 2024 Jul 29;19(7):e0306126. doi: 10.1371/journal.pone.0306126 (PMC11285961; doi:10.1371/journal.pone.0306126)
Supplement: S1 File — (DOCX) [file pone.0306126.s001.docx]

**Interview guide**

***Introductory question:***

Could you tell us about your experiences of the sick leave and rehabilitation process?

***Research questions:***

1. Could you tell us about your experiences of what affects the outcome of the sick leave and rehabilitation process?
2. Could you tell us about your experiences of organizational support to apply best practices in the sick leave and rehabilitation process?

*How would you describe the organizational support for tailoring process activities to patients’ needs?*

*How would you describe collaboration and consensus in the sick leave and rehabilitation process, and what are the perceived needs?*

1. Could you tell us about potential sick leave and rehabilitation process improvements that you identify based on your experiences?

*How, according to your experiences, could your profession (or role) contribute to an improved process?*

1. What are your experiences of continuous improvement work in the sick leave and rehabilitation process?

*What are your experiences of improvement regarding work coordination between the different actors?*

*How do you perceive your opportunities to contribute to process improvements?*
