# Supplementary material for: Professionals’ experiences of what affects health outcomes in the sick leave and rehabilitation process—A qualitative study from primary care level
Source: PLoS One. 2024 Jul 29;19(7):e0306126. doi: 10.1371/journal.pone.0306126 (PMC11285961; doi:10.1371/journal.pone.0306126)
Supplement: Attachment — Submitted filename: Response to Reviewers 240423.docx [file pone.0306126.s002.docx]

**Response to reviewers and members of the Editorial Board**

Firstly, we would like to thank you very much for the time and effort you have invested in revising our manuscript! We are very grateful for your insightful feedback, which we find greatly improves the manuscript. Below follows a point-by-point response including our considerations and changes related to the suggestions raised. We very much hope our changes will meet your expectations.

Per your instructions, you will find our manuscript in an annotated copy, using the Track Changes function in Word to highlight changes made to the original version, and an unmarked version of our revised paper without tracked changes uploaded to the website.

**Abstract**

***Comment 1.*** *”Professional actors could be a confusing term, the word actors could be misinterpreted.”*

Response: Thank you for making us aware of the term ”professional actors” being confusing. ”Professional actors” refers to frontline employees, with different professional backgrounds, working in direct contact with patients within the sick leave and rehabilitation process (SRP). We chose the term ”professional actors” to underscore the professional expertise of the participants. Considering your comment we decided to use the term ”frontline employees” throughout the article, and in the tables.

***Comment 2 and 3.*** *”Include an explanation of why these particular professionals were recruited.” & ”Describe what type of analysis that was used.”*

Response: We acknowledge that we should have mentioned an explanation of why the particular professionals were recruited in the abstract or the type of analysis used. In line with your feedback, the description of the method in the abstract has been changed to:

”Qualitative design based on focus group interviews in a primary care context in Region Västra Götaland, Sweden. Strategically selected professionals from different SRP organizations discussed sick leave outcomes and the rehabilitation process. Analysis was performed with Systematic text condensation.”

(To limit the abstract word count, ”conscious and unconscious bio-psycho-social needs” was changed to ”bio-psycho-social needs”.)


**Method**

***Comment 4.*** *”Describe why this method was suitable for this study, why was it selected instead of other qualitative approaches?”*

Response: Thank you for this input. Based on your comment, we have added the following to the description of data analysis in the methods section on pages 11-12:

”This qualitative analysis method was chosen because of its pragmatic, holistic, explorative, and reflexive approach, which we believe suited our overall aim of presenting decision support for managing quality-driven change management within SRP, considering the perspectives of different professional parties involved in SRP, and our holistic approach.”

***Comment 5.*** *”Did you reflect on differences in the interviews between digital and physical focus groups?”*

Response: Thank you for pointing out the necessity of reflecting on the impact of digital and physical focus groups. Following this comment, we have added a reflection to ”Strengths and limitations” in the discussion on page 29:

”Digital and physical focus groups were equally rich in content, lively, and engaged. There was a minor difference in the quality of data collected overall. Nevertheless, we noted that it was much more strenuous for the interviewers to conduct the digital focus groups, as the lack of direct interaction between people required the interviewers’ compensatory effort."

***Comment 6.*** *”How was the interview guide developed?”*

Response: In the focus groups, the interviewees only needed the introductory question to initiate vivid discussions. Therefore, the interview guide was used more as a checklist than was initially intended. This comment has been addressed by adding a sentence to ”Data collection” in the methods section on page 11:

”As the interviewees only needed the introductory question to initiate vivid discussions, the interviewers used the interview guide to make sure all four research questions were addressed. Still, the questions were addressed in different orders in the various focus groups depending on how the discussions went in each group.”

***Comment 7.*** *”The research questions are presented for the first time under the section analysis. Why are these research questions not presented earlier? And how were they developed in relation to the research aim?”*

Response: Thank you for drawing our attention to the fact that the research questions are presented in the section analysis and the very relevant need to clarify how these questions relate to the research aim in this specific study.

We have clarified that this study was from the start part of a larger holistic project aiming to answer four predefined research questions. We have also moved the presentation of the predefined research questions (matching questions 1-4 in the interview guide) to the description of the study design in the methods section on page 7, and clarified that the present study aim corresponds to answering only the first predefined question: *”What are SRP frontline employees' experiences of what affects SRP outcome?”*.

”This study is part of a larger holistic project with the overall aim of presenting decision support for policymakers by answering four predefined research questions: ”What are SRP frontline employees' experiences of what affects SRP outcome?”, ”What are SRP frontline employees’ experiences of organizational support to apply best practices in SRP?”, ”What potential process improvements do the SRP frontline employees identify based on their experiences?”, and ”What are SRP frontline employees’ experiences of continuous improvement work in the SRP?”. This particular study, with its specific background, analysis, discussion, and conclusions, focuses only on answering the first research question.”

Further, we have clarified that data collection, to answer the four predefined questions that together formed the larger holistic project, was carried out jointly. See the description of the study design in the methods section on page 7.

”Data collection was carried out jointly to answer the four predefined research questions, using digital and physical focus group discussions (either/or, no blended groups).”

**Comment 8. *”****In the article it is stated: “ Therefore, the research group decided to report the analysis in a series of three different articles.” This is unclear to me, will the same analysis be presented in three different articles? If so, I think that is a problem. The data consists of six interviews. In my view this is not a lot of data to justify different articles. And how are the findings presented in this article related to the findings in the other two articles? I suggest that the analysis form the six interviews is presented in this article.”*

Response: Thank you for this very important question, which makes us aware that we have not sufficiently explained why there is a need for three separate articles.

The three articles that together compose the larger project are based on three separate analysis processes that each circled the different predefined research questions: #1 (this article), #2 (the second article), and #3-4 (the third article). Neither study aims, nor analysis code groups or subgroups overlap. Further, different theoretical frameworks are presented in the introductions, and the findings are interpreted by comparing with different research fields. Also the conclusions and implications of the findings differ.

The collected data encompassed nearly 12 hours of focus group interviews with rich content and lively discussions with input from different professions and different organizations. When transcribed the data comprised 415 pages (Times New Roman, 12 p, double line spacing).

We knew from the start that answering all predefined research questions would require separate analyses. Still, we did not know if the answers to the four predefined research questions would overlap, and we had yet to anticipate the richness of the collected data. Therefore, we had yet to decide on the number of articles beforehand.

In response to your comment, we have refined the description of the data analysis to deal only with the article in question by omitting the unclear passage about ”reporting the analysis in a series of three different articles”. We believe the clarifications described in response to ***Comment 7.***, presented in the study design section on page 7, sufficiently explain the joint data collection, the relation between the predefined research questions of the overall project, and the relation to the research question of the present study.

However, as the three articles are not overlapping and the collected data very well justify them, the most logical solution is to present the empirical data separately. We hope you will consider this decision reasonable based on the above explanation. Otherwise, please do not hesitate to contact us with further questions.

**Results**

***Comment 9.*** *”What do you mean by ‘meets patients conscious and unconscious needs? What needs are unconscious, and who have been able to identify these needs? I think the word unconscious is problematic. Could it be explained in a different way?”*

Response: Thank you for this comment, which made us aware of the need for clarifications. ”Patients' conscious and unconscious needs” refers to needs that patients are aware of and, therefore, spontaneously express and the additional needs that the interviewees, with their professional expertise, may identify and address in dialogue with the patients. We have clarified this in the result section on page 13.

***Comment 10 & 11.*** *”I would suggest that you use headings in your result section to make clear what code group and you are presenting.” & ”The subgroups are not always clear in the analysis. I would suggest that you revise the analysis so that the subgroups can be easily found in the result section. Make it very easy for the reader to follow and understand your results.”*

Response: Thank you very much for this advice; it helped structure the results section. We have added headings for the subgroups in the results section. In doing so, we realized that you were right when commenting on the subgroups needing to be more apparent in the analysis. We have therefore revised the analysis so that the subgroups can be more easily found. The process entailed two cases of lumping subgroups, thus also shortening the results section. See the results section and Table 2.

**Discussion**

***Comment 12.*** *”The fist paragraph in the discussion presents what need to be done to optimize outcomes form SRP. Are these the same as your code groups, or are they somewhat different? For example Early ‘early bio-psycho-social assessments’ appears to be one sub group in the first code group (or I may have misunderstood this). It would make it more easy to understand if you connect the summary of your results to your code groups.”*

Response: Thank you for this comment. We acknowledge the need to clarify the first paragraph of the discussion to match the code groups more clearly. We have rewritten the first paragraph of the discussion on page 23 to more clearly summarize the code groups and their respective subgroups to increase comprehensibility.

”Our results indicate that to optimize outcomes from SRP, the process needs to embrace and facilitate the management of both medical and non-medical factors affecting health and work capacity by incorporating the following aspects: 1) creating consensus about causes of ill-health and opportunities available through early bio-psycho-social assessments, including medical specialist consultations when needed, 2) a realistic rehabilitation plan for regaining and sustaining health through timely multi-professional rehabilitation, practical support to overcome obstacles in daily life, and RTW, alongside medical treatment, 3) access to a wide range of rehabilitative and supportive interventions to enable participation in individually meaningful activities and preserving skills, and 4) trusting relationships over time to maximize process quality and person-centeredness.”

***Comment 13.*** *”The first main finding presented is not one of the code groups. This is a bit confusing, or is it Is this relating to the first code group, creating consensus?”*

Response: Thank you for this valuable comment, which makes us aware of the need to relate the discussion more clearly to the code groups. (As you suggested, the paragraph “A main finding of this article was that the interviewees, regardless of their profession and role, strongly agreed on the perception that reduced work capacity rarely can be explained solely by medical diagnoses” relates to the first code group, creating consensus.)

We have omitted the numbering of the main findings and clarified which parts of the discussions belong to which theme (code group). We have also rearranged the discussion section on pages 23-28 so that the themes are discussed in the same order as in the results section.

***Comment 14.*** *”There are very few limitations included. Could it be a limitation that some of the interviews were digital and some live? I would suggest that the authors reflect on this under limitations.*

Response: Thank you for pointing out this. We have added a passage about digital and physical focus groups and a paragraph about the need to utilize qualitative findings wisely as they are based on small, strategically selected samples. See strengths and limitations in the discussion on pages 29-30.

”Digital and physical focus groups were equally rich in content, lively, and engaged. There was a minor difference in the quality of data collected overall. Nevertheless, we noted that it was much more strenuous for the interviewers to conduct the digital focus groups, as the lack of direct interaction between people required the interviewers’ compensatory effort.

Unlike quantitative research, qualitative findings do not prove hypotheses, as participant selection is strategic rather than random, and conclusions are drawn from the statements of a select few individuals. Instead, qualitative research methods aim to uncover the feelings, beliefs, and experiences of a particular target group regarding a specific topic, providing insight into their behaviors. When utilized thoughtfully, the descriptive findings from qualitative research play a critical role in making contextually relevant decisions and laying the groundwork for subsequent quantitative studies.”

***Comment 15.*** *Reviewer #2: ”The manuscript was well written and informative, the readers will be able to learn a methodology of a qualitative study. If possible to shorther the lenght of manuscript the results may be shorthen.”*

Response: Thank you for your kind words about our study. We have revised the analysis to more clearly separate the subgroups, which entailed two cases of lumping subgroups. See also Comment 10 & 11, the results section pages 13-22, and table 2.

***Comment 16.*** *”The interpretation of results may be added to the discussion no need to have a separated section and more focust and expamd the link with SRP in order to highlight the application of the result.”*

Response: Thank you for this comment. It makes us aware of the need to relate our findings more clearly to the SRP.

We have omitted the subheading *Interpretation of results and comparison with previous work.* Further, we have structured the discussion section to more clearly relate to the themes of the results section, and the SRP. See Comment 13, and the discussion paragraphs 2-13 on pages 23-28.
